# Supplementary material for: Mapping Change in Large Networks
Source: PLoS One. 2010 Jan 27;5(1):e8694. doi: 10.1371/journal.pone.0008694 (PMC2811724; doi:10.1371/journal.pone.0008694)
Supplement: Appendix S1 — Here we briefly review our information theoretic approach to revealing community structure in weighted and directed networks and present a new fast stochastic and recursive search algorithm to minimize the map equation — the objective function of our method. (0.14 MB PDF) [file pone.0008694.s001.pdf]

Appendix S1

M. Rosvall*

Department of Biology, University of Washington, Seattle, WA 98195-1800†

C. T. Bergstrom‡

Department of Biology, University of Washington, Seattle, WA 98195-1800† *and*
Santa Fe Institute, 1399 Hyde Park Rd., Santa Fe, NM 87501§

(Dated: December 18, 2009)

Mapping directed weighted networks

Here we briefly review our information theoretic approach to revealing community structure in weighted and directed networks [1] and present a new fast stochastic and recursive search algorithm to minimize the map equation — the objective function of our method. We have developed this algorithm to be able to accurately partition the large number of bootstrap networks [2]. The search algorithm can also be generalized for other objective functions.

The map equation

The objective of our flow-based and information-theoretic method known as the map equation is to find the structures within a network that are significant with respect to how information or resources flow through that network. For a detailed description of the map equation, see ref. [3]. For a dynamic visualization of the mechanics of the map equation, see http://www.tp.umu.se/~rosvall/livemod/mapequation/. The following is a short review.

There is a duality between the problem of compressing a data set, and the problem of detecting and extracting significant patterns or structures within those data [4–6]. We have developed the map equation approach to make use of this duality to detect community structure within directed and weighted networks that inherently are characterized by flow. For a given network partition M, the map equation specifies the theoretical limit $L(\mathsf{M})$ of how concisely we can describe the trajectory of a random walker on the network. The underlying code structure of the map equation is designed such that the description can be compressed if the network has regions in which the random walker tends to stay for a long time. Therefore, with a random walk as a proxy for real flow, minimizing the map equation over all possible network partitions reveals important aspects of network structure with respect to the dynamics on the network.

To take advantage of the regional structure of the network, one index codebook and m module codebooks, one for each module in the network, are used to describe the random walker's movements. The module codebooks have codewords for nodes within each module (and exit codes to leave the module), which are derived from the node visit/exit frequencies of the random walker. The index codebook has codewords for the modules, which are derived from the module switch rates of the random walker. Therefore, the average length of the code describing a step of the random walker is the average length of codewords from the index codebook and the module codebooks weighted by their rates of use. This is the map equation:

$$L(\mathsf{M}) = q_{\curvearrowright} H(\mathcal{Q}) + \sum_{i=1}^{m} p_{\circlearrowright}^{i} H(\mathcal{P}^i). \qquad (1)$$

The first term of this equation gives the average number of bits necessary to describe movement between modules, and the second term gives the average number of bits necessary to describe movement within modules. In the first term, $q_{\curvearrowright}$ is the probability that the random walker switches modules on any given step, and $H(\mathcal{Q})$ is the entropy of the module names, i.e. the frequency-weighted average length of codewords in the index codebook. In the second term, $H(\mathcal{P}^i)$ is the entropy of the within-module movements — including an "exit code" to signify departure from module i, i.e. the frequency-weighted average length of codewords in module codebook i — and the weight $p_{\circlearrowright}^{i}$ is the fraction of within-module movements that occur in module i, plus the probability of exiting module i such that $\sum_{i=1}^{m} p_{\circlearrowright}^{i} = 1 + q_{\curvearrowright}$.

To efficiently describe a random walk using a two-level code of this sort, the choice of partition M must reflect the patterns of flow within the network, with each module corresponding to a cluster of nodes in which a random walker spends a long period of time before departing for another module. To find the best such partition, we therefore seek to minimize the map equation over all possible partitions M.

*Electronic address: rosvall@u.washington.edu
†URL: http://www.tp.umu.se/~rosvall
‡Electronic address: cbergst@u.washington.edu
§URL: http://octavia.zoology.washington.edu/

Fast stochastic and recursive search algorithm

Any greedy (fast but inaccurate) or Monte Carlo-based (accurate but slow) approach can be used to minimize the map equation. To provide a good balance between the two extremes, we have developed a fast stochastic and recursive search algorithm, implemented it in C++, and made it available online both for directed and undirected weighted networks [2]. As a reference, the new algorithm is as fast as the previous high-speed algorithms (the greedy search presented in the supporting appendix of ref. [1]), which were based on the method introduced in ref. [7] and refined in ref. [8]. At the same time, it is also more accurate than our previous high-accuracy algorithm (a simulated annealing approach) presented in the same supporting appendix.

The core of the algorithm follows closely the method presented in ref. [9]: neighboring nodes are joined into modules, which subsequently are joined into supermodules and so on. First, each node is assigned to its own module. Then, in random sequential order, each node is moved to the neighboring module that results in the largest decrease of the map equation. If no move results in a decrease of the map equation, the node stays in its original module. This procedure is repeated, each time in a new random sequential order, until no move generates a decrease of the map equation. Now the network is rebuilt, with the modules of the last level forming the nodes at this level. And exactly as at the previous level, the nodes are joined into modules. This hierarchical rebuilding of the network is repeated until the map equation cannot be reduced further. Except for the random sequence order, this is the algorithm described in ref. [9].

With this algorithm, a fairly good clustering of the network can be found in a very short time. Let us call this the core algorithm and see how it can be improved. The nodes assigned to the same module are forced to move jointly when the network is rebuilt. As a result, what was an optimal move early in the algorithm might have the opposite effect later in the algorithm. Because two or more modules that merge together and form one single module when the network is rebuilt can never be separated again in this algorithm, the accuracy can be improved by breaking the modules of the final state of the core algorithm in either of the two following ways:

> *Submodule movements.* First, each cluster is treated as a network on its own and the main algorithm is applied to this network. This procedure generates one or more submodules for each module. Then all submodules are moved back to their respective modules of the previous step. At this stage, with the same partition as in the previous step but with each submodule being freely movable between the modules, the main algorithm is re-applied.
>
> *Single-node movements.* First, each node is re-assigned to be the sole member of its own module, in order to allow for single-node movements. Then all nodes are moved back to their respective modules of the previous step. At this stage, with the same partition as in the previous step but with each single node being freely movable between the modules, the main algorithm is re-applied.

In practice, we repeat the two extensions to the core algorithm in sequence and as long as the clustering is improved. Moreover, we apply the submodule movements recursively. That is, to find the submodules to be moved, the algorithm first splits the submodules into subsubmodules, subsubsubmodules, and so on until no further splits are possible. Finally, because the algorithm is stochastic and fast, we can restart the algorithm from scratch every time the clustering cannot be improved further and the algorithm stops. The implementation is straightforward and, by repeating the search 100 times, the final partition is less likely to correspond to a bad clustering of a local minimum. For each iteration, we record the clustering if the description length is shorter than the previously shortest description length. In practice, for the citation networks presented in this paper, which have on the order of 10,000 nodes and 1,000,000 directed and weighted links, each iteration takes about 5 seconds on a modern PC. We generate the significance clusterings by repeating the algorithm 100 times for each network and bootstrap network.

References

[1] Rosvall M, Bergstrom CT (2008) Maps of information flow reveal community structure in complex networks. *Proc Natl Acad Sci USA* 105:1118–1123.

[2] The code can be downloaded from http://www.tp.umu.se/~rosvall/code.html.

[3] Rosvall M, Axelsson D, Bergstrom C (2009) The map equation. arXiv:0906.1405.

[4] Shannon CE, Weaver W (1949) *The mathematical theory of communication* (Univ of Illinois Press).

[5] Rissanen J (1978) Modeling by shortest data description. *Automatica* 14:465–471.

[6] Grünwald P, Myung IJ, Pitt M, eds (2005) *Advances in minimum description length: theory and applications* (MIT Press, London, England).

[7] Clauset A, Newman MEJ, Moore C (2004) Finding community structure in very large networks. *Phys Rev E* 70:066111.

[8] Wakita K, Tsurumi T (2007) Finding community structure in mega-scale social networks. arXiv:cs/07020480.

[9] Blondel V, Guillaume J, Lambiotte R, Mech E (2008) Fast unfolding of communities in large networks. *J Stat Mech: Theory Exp* 2008:P10008.
