# Supplementary material for: Mapping Change in Large Networks
Source: PLoS One. 2010 Jan 27;5(1):e8694. doi: 10.1371/journal.pone.0008694 (PMC2811724; doi:10.1371/journal.pone.0008694)

Mapping change in physics & chemistry 1997–2007

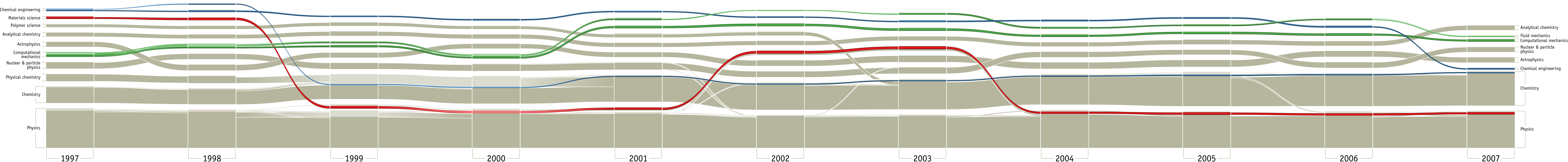

Supplement: Figure S2 — Mapping change in physics & chemistry 1997–2007 (0.06 MB PDF) [file pone.0008694.s003.pdf]
